# Nutritional Composition, Antioxidant Activity, and Phytochemical Analysis of Three Commonly Consumed Wild Edible Plants in Dibatie District, Western Ethiopia

**DOI:** 10.1155/tswj/6658147

**Published:** 2025-11-17

**Authors:** Baressa Anbessa, Ermias Lulekal, Ariaya Hymete, Paulos Getachew

**Affiliations:** ^1^Department of Plant Biology and Biodiversity Management, College of Natural and Computational Sciences, Addis Ababa University, Addis Ababa, Ethiopia; ^2^Department of Biology, College of Natural and Computational Sciences, Bule Hora University, Bule Hora, Ethiopia; ^3^Department of Pharmaceutical Chemistry and Pharmacognosy, School of Pharmacy, College of Health Sciences, Addis Ababa University, Addis Ababa, Ethiopia; ^4^Center for Food Science and Nutrition, College of Natural and Computational Sciences, Addis Ababa University, Addis Ababa, Ethiopia

**Keywords:** antinutritional, antioxidant, *D. praehensilis*, nutritional value, phytochemical, *S. comorensis*, *S. guineense* subsp. *macrocarpum*

## Abstract

**Background:**

Wild edible plants supplement households' food supply as seasonal or emergency foods in different communities of Ethiopia. However, local people consume them without considering their nutritional values.

**Objective:**

This study is aimed at evaluating the nutritional value, antinutritional, antioxidant, and phytochemical profiles of *Saba comorensis* (Bojer ex A.DC.) Pichon fruits, *Syzygium guineense* (Wild.) DC. subsp. *macrocarpum* (Engl.) F. White fruits, and *Dioscorea praehensilis* Benth. tubers consumed in Dibatie district, western Ethiopia.

**Methods:**

Juices of edible plants were used to determine pH, acidity, and total soluble solid. Lyophilized powders were analyzed to determine vitamin C, proximate composition, minerals, antinutritional factors, antioxidant capacity, and phytoconstituents.

**Results:**

The *S. comorensis* fruits had the highest (*p* < 0.05) acidity, vitamin C, and total soluble solid. The studied plants contained 2.50%–15.50% crude fiber, 0.75%–4.00% crude fat, 4.38%–10.50% crude protein, 59.63%–68.83% carbohydrate, and 267.75%–324.08 kcal/100 g energy. The studied plants had minerals like calcium (522.27–995.04 mg/100 g), iron (19.80–111.94 mg/100 g), magnesium (923.25–1592.18 mg/100 g), manganese (0.50–5.72 mg/100 g), potassium (591.69–1357.71 mg/100 g), sodium (0.60–17.17 mg/100 g), and zinc (1.00–1.74 mg/100 g). The tested plants had 65.11–70.67 mg/100 g phytates, 170.00–790.00 mg/100 g oxalates, and 196.51–11147.55 mg CE/100 g tannins. *S. comorensis* fruits showed substantial DPPH free radical scavenging activity with an IC_50_ value of 0.07 mg/mL, because they were significantly (*p* < 0.05) the highest in phenolics, flavonoids, and alkaloids.

**Conclusion:**

The investigated plants were rich suppliers of valuable macro- and micronutrients and phytochemicals, along with considerable antinutritional and antioxidant properties. Thus, they require special conservation and management measures for sustainable usage by the local communities and Ethiopian people as a whole.

## 1. Introduction

The consumption of wild edible plants (WEPs) is a fundamental culture in different rural communities of Ethiopia [1]. They serve as additional, seasonal, and emergency aids for household food supplies [2]. The issue of food insecurity is resolved by a wider usage of WEPs in certain drought-prone parts of the country [3]. They can be used as chief sources of food both during an abundance of regular food and during food scarcity [4]. Some WEPs are consumed only when preferred alternatives do not exist or during a serious food shortage [5]. Because their consumption is usually considered an implication of poverty in the majority of Ethiopian communities [6], there is a lack of awareness of their nutritional values and health effects, which are comparable to those of cultivated plants [7].

However, indigenous WEPs are good sources of nutrients such as protein, crude fiber, carbohydrate, moisture, vitamins A and C, and essential minerals like calcium, iron, magnesium, manganese, zinc, and phosphorous [6–8]. For example, WEPs such as *Celosia argentea* L., *Coccinia grandis* (L.) Voigt, *Cynanchum insipidum* (E.Mey.) Liede & Khanum, and *Justicia flava* (Forssk.) Vahl are reported to be rich in protein content [6]. They also play an important role in improving human health because of their therapeutic properties [9]. In this regard, they are considered “nutraceutical plants” because of their both medicinal and nutritional advantages [10]. For instance, wild edible indigenous plants are potential sources of novel natural products [11], which help with weight management and minimize health problems like cardiovascular diseases [12] since they constitute various phytochemicals, including phenolic compounds and flavonoids [13].

Moreover, some WEPs (e.g., *Saba comorensis* [Bojer ex A.DC.] Pichon and *Vitex doniana* Sweet) were reported to have even more antioxidant activities than certain cultivated plants such as *Ananas comosus* (L.) Merr., *Citrus × aurantium* f. *aurantium*, *Citrus × limon* (L.) Osbeck, and *Musa acuminata* Colla [14]. For agrofood industries and gastronomy, consumers currently need more natural additives than synthetic ones [15]. Plant-derived natural bioactive compounds are essential sources of lead compounds in the pharmaceutical industry for drug development [16]. Consequently, a large number of WEP extracts are safe, and they may be used as antioxidant supplements and additions to treat oxidative stress–related illnesses [17].

Nevertheless, edible wild or semiwild plants may constitute natural antinutritional substances that prevent the absorption of essential nutrients, similar to some cultivated green vegetables [6]. Antinutritional substances like phytate and oxalate limit the absorption of essential minerals by binding with metal ions like calcium, iron, magnesium, and zinc [18, 19]. Besides, the interaction of factors such as protease inhibitors, amylase inhibitors, phytates, oxalates, lectins, goitrogens, hydrogen cyanide, total free phenolics, and tannins influences protein digestion [20]. As a result, appropriate measures (processing technologies) are required to reduce their levels in wild edible fruits and vegetables [6]. The quantity of antinutritional components in edible plants can be minimized by using food preparation techniques like fermentation, heating, soaking, and puffing [19].

*S. comorensis* (Bojer ex A.DC.) Pichon (locally called *Wenno*), *Syzygium guineense* (Wild.) DC. subsp. *macrocarpum* (Engl.) F. White (locally named *Goosu*), and *Dioscorea praehensilis* Benth. (locally called *Eeca*) are wild plants eaten by local residents of Dibatie district, western Ethiopia [21]. The fruits of *S. comorensis* are harvested from late May to late June by youth, students, and cattle herders. The fruits of *S. guineense* subsp. *macrocarpum* are eaten by people of any age group, predominantly in April and May. In addition, tubers of *D. praehensilis* are consumed, roasted, or boiled as supplementary food by people of any age group almost throughout the year. These plants are widely consumed by the local communities, mainly as a supplementary food or to fill the seasonal food gap, without considering their nutritional values, phytochemical contents, or health effects. Besides, except for the studies conducted on the nutritional composition of *D. praehensilis* tubers in Ethiopia [22] and on the antioxidant activity of *S. comorensis* fruits in Tanzania [14], no previous research has documented the nutritional, antinutritional, antioxidant, and phytochemical properties of these WEPs to date. Therefore, the current study is aimed at evaluating the nutritional, antinutritional, antioxidant, and phytochemical constituents of the abovementioned WEPs, which are commonly consumed by the indigenous people in the Dibatie district of the Metekel zone, western Ethiopia.

## 2. Materials and Methods

### 2.1. Sample Collection and Preparation

**Figure 1:**
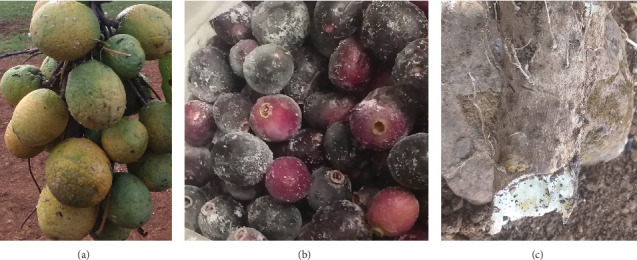
Edible parts of the studied plants. (a) *S. comorensis* fruits, (b) *S. guineense* subsp. *macrocarpum* fruits, and (c) *D. praehensilis* tubers.

The ripe fruits of *S. comorensis* and *S. guineense* subsp. *macrocarpum*, and the tubers of *D. praehensilis* (Figure 1), were collected in May and June 2022. The sample collection was carried out from Lega-buna (10° 36.166⁣′ N and 36° 09.509⁣′ E), Jan (10° 33.603⁣′ N and 36° 07.959⁣′ E), and Gipho (10° 28.340⁣′ N and 36° 06.110⁣′ E) subdistricts (kebeles) of the Dibatie district, western Ethiopia. The collected samples were brought to the Center for Food Science and Nutrition Laboratory of Addis Ababa University. The edible, fleshy mesocarp of *S. comorensis* fruits was separated manually from the seeds and exocarp and kept in a clean polythene bag at −20°C. The edible, fleshy exocarp of *S. guineense* subsp. *macrocarpum* fruits was peeled off manually and preserved in a clean polythene bag at −20°C, while the inner stony seeds were discarded. Tubers of *D. praehensilis* were peeled, sliced with a stainless steel knife, and stored in a polythene bag at −20°C. The edible parts were stored in a deep freeze for about 1–2 days until lyophilizing. The fresh and/or dissolved juices of edible parts were applied for the determination of pH value, titratable acidity, and total soluble solid (TSS). The edible parts were lyophilized, pulverized into a fine powder, and preserved in deep freeze until analysis of vitamin C, proximate composition, minerals, antinutritional, antioxidant, and phytochemical constituents.

### 2.2. Extraction of Plant Materials

The plant materials were extracted and stored following the procedures stated by Muddathir et al. [23]. Briefly, the lyophilized powder (5 g) was macerated in 50 mL of absolute methanol (99.8%) with continuous shaking for 24 h. Then, the mixture was filtered using Whatman No. 1 filter paper, and the residue was macerated in the same solvent again, followed by filtration. After combining the filtrates, the solvent was removed in vacuo using a rotary evaporator set at 40°C. Until being utilized for analysis, every dried extract was kept at 4°C.

### 2.3. Determination of pH and Titratable Acidity

The pH value was determined following the procedures stated by Korese et al. [24], with a few minor adjustments. That is, a frozen 20 g of each sample was dissolved in 20 mL of distilled water and well homogenized. Then, a digital pH meter was used to determine the pH value of homogenized samples. The pH meter was maintained at 20.60°C and calibrated using standard buffer solutions with pH values of 4.0 and 7.0. Later, the electrode was cleaned with distilled water and wiped dry with tissue paper before the pH determination for each sample.

The titratable acidity of the edible samples was determined following the approaches used by Sharma and Saini [25] and Tiencheu et al. [26]. In short, 5 g of each sample was dissolved in 20 mL of distilled water, followed by filtration using Whatman No. 1 filter paper. To attain a pH of 8.1, a known volume (10 mL) of the filtrate was titrated against 0.1 N NaOH by recording the consumed volume of sodium hydroxide. The total titratable acidity was calculated by using the formula:

(1) $$\begin{array}{c} \%\text{Acid}=\frac{\left(\text{volume of base titrant }\right)\times \left(\text{normality of base}\right)\times \left(\text{Eq}.\text{wt}.\text{of acid}\right)}{\left(\text{volume of sample}\right)\times 10}, \end{array}$$

where Eq. wt. of acid is equivalent weight of citric acid (64.04) [27].

### 2.4. Determination of TSSs and Vitamin C

The TSSs of the fresh fruit juice were measured using digital refractometers and expressed as “degrees of Brix” (°Brix). Vitamin C was determined spectrophotometrically by using potassium permanganate, following Bayang et al. [28]. The lyophilized sample powder (0.2 g) was combined with 5 mL of 0.5% oxalic acid, agitated in the dark for 24 h, and filtered using Whatman No. 1 filter paper. Then, 0.5 mL of sample was thoroughly combined with 1.5 mL of 0.1% KMnO_4_, which was previously dissolved in 0.1 M H_2_SO_4_. The absorbance was measured at 525 nm with a UV–Vis spectrophotometer. Vitamin C was determined using the standard L-ascorbic acid (0–80 *μ*g/mL) calibration curve. Results were quantified as milligrams of L-ascorbic acid equivalent per 100 g of sample.

### 2.5. Proximate Composition Analysis

The composition of the study plants in terms of moisture, total ash, crude fiber, crude protein, and crude fat was measured using the Association of Official Analytical Chemists [29]. The moisture content was ascertained by drying in an oven (OV/125/SS/F/DIG/A, Genlab Limited, United Kingdom) at 105°C until a constant weight was obtained. Then, the percent of moisture content was calculated using the formula:

(2) $$\begin{array}{c} \%\text{Moisture }\left(\frac{W}{W}\right)=\frac{\left(\text{weight of wet sample}-\text{weight of dry sample}\right)}{\text{weight of wet sample}}\times 100. \end{array}$$

Ash content was determined using a heated muffle furnace (Furnace type CSF 12/13, Carbolite, England) at 550°C. The percent of ash content was calculated by using the formula:

(3) $$\begin{array}{c} \%\text{Ash }\left(\text{dry basis}\right)=\frac{\text{ash weight}}{\text{dry sample weight}}\times 100. \end{array}$$

Crude fiber content was ascertained following the AOAC Official Method 920.86 by digesting fat-free samples with 1.25% sulfuric acid and 1.25% sodium hydroxide solutions, followed by the weight loss on ignition of dried residue. The percentage of crude fiber content was calculated by using the equation:

(4) $$\begin{array}{c} \text{Crude fiber }\left(\%\right)=\frac{W1-W2}{W}\times 100, \end{array}$$

where *W*1 is the weight of dry residue before ashing, *W*2 is the weight of residue after ashing, and *W* is the weight of the sample.

Protein quantification was carried out by the Kjeldahl method (BUCHI Distillation Unit K-350, Switzerland), depending on the levels of nitrogen. The percentage of nitrogen was calculated first by the formula:

(5) $$\begin{array}{c} \%\text{Nitrogen}=\text{normality of HCl}\times \frac{\text{corrected acid volume }\left(\text{mL}\right)}{\text{g of sample}}\times \frac{14\text{ g nitrogen}}{\text{mol}}\times 100. \end{array}$$

Then, the percentage of protein was obtained by multiplication of the % nitrogen with protein factor (6.25).

Fat was quantified using the full automated Soxhlet (BSXT-06 Soxhlet Extractor, Wuhan, China) extraction using petroleum ether. Then, the percentage of fat was calculated using the formula:

(6) $$\begin{array}{c} \%\text{Fat }\left(\text{dry basis}\right)=\frac{\text{weight of extracted fat}}{\text{weight of dry sample}}\times 100. \end{array}$$

The total carbohydrate content was calculated from the difference using the following equation:

(7) $$\begin{array}{c} \text{Carbohydrate }\left(\%\right)=100-\left(\text{moisture}+\text{ash}+\text{crude fiber}+\text{crude protein}+\text{crude fat}\right)\%. \end{array}$$

The gross calorific value of the edible samples was calculated using the following equation:

(8) $$\begin{array}{c} \text{Gross energy }\left(\frac{\text{kcal}}{100}\text{g}\right)=4\times \text{protein }\left(\%\right)+4\times \text{carbohydrate }\left(\%\right)+9\times \text{fat }\left(\%\right). \end{array}$$

### 2.6. Determination of Minerals

The mineral composition was quantified following the method described in Hegazy et al. [30]. The concentration of mineral ions such as calcium, copper, magnesium, manganese, iron, and zinc was quantified using atomic absorption spectrophotometry (AA 800, PerkinElmer, Canada) after wet digestion. A microwave plasma atomic emission spectrometer (4210 MP-AES, Agilent, United States) was used to measure the sodium and potassium contents. Phosphorus was determined colorimetrically by a UV–Vis spectrophotometer (Lambda 950, PerkinElmer, Waltham, United States) using the potassium dihydrogen phosphate standard.

### 2.7. Determination of Antinutritional Factors

Phytic acid determination was performed using Wade's reagent colorimetric technique used in Achaglinkame et al. [31]. Triplicate sample powder (0.2 g) was extracted in 10 mL of 0.2 M HCl for 1 h, followed by centrifugation for 30 min at 1790 g. The supernatant (3 mL) was transferred into a clean test tube along with Wade's reagent (2 mL) (i.e., sulfosalicylic acid and FeCl_3_.6H_2_O solution in a 1:1 ratio) and 0.2 M hydrochloric acid (2 mL). A UV–Vis spectrophotometer was used to measure the absorbance of the mixture at 500 nm. The phytic acid standard curve was employed to calculate the phytate content.

The determination of oxalic acid was carried out by colorimetric titration using potassium permanganate, as stated in Djikeng et al. [32]. Briefly, each sample powder (1 g) was mixed with 75 mL of 3 M H_2_SO_4_, agitated carefully using a magnetic stirrer for an hour, followed by filtration with Whatman No. 1 filter paper. The filtrate (25 mL) was collected and titrated until a faint pink color that lasted for 30 s was achieved against a heated (80°C–90°C) 0.1 M KMnO_4_ solution. The level of oxalic acid in each sample was calculated from the equation: 1 mL of 0.1 M KMnO_4_ consumption = 0.00450 g of oxalic acid in the sample. The experiment was done in triplicates for each sample.

The tannic acid was quantified using the vanillin–HCl assay technique employed in Wu et al. [33]. Each sample powder (1 g) was measured in a test tube and combined with 1% of hydrochloric acid (10 mL) in methanol. The mixture was shaken mechanically for 24 h, followed by centrifugation for 5 min at 1790 g. In a separate test tube, 1 mL of the supernatant was combined with 5 mL of the vanillin–HCl reagent. After letting the solution stand for 20 min, the absorbance was measured using UV–Vis spectrophotometry at 500 nm. Catechin standard reference was used, and the final value was expressed as milligrams of catechin equivalents (mg CE) per gram sample. The experiment was done in triplicates for each sample.

### 2.8. Determination of Antioxidant Activity

The 2,2-diphenyl-1-picrylhydrazyl (DPPH) free radical scavenging assay was assessed spectrophotometrically, as previously stated in Amari et al. [34]. For each sample, a 50 mg/mL stock solution of crude extract was made in methanol. A solution of DPPH was mixed with extracts in methanol at different concentrations (0.02, 0.04, 0.08, 0.12, 0.16, and 0.2 mg/mL). After gently vortexing the mixture, it was allowed to keep in the dark for half an hour. Using a UV–Vis spectrophotometer, the absorbance of a mixture was read against a blank at 517 nm. The experiment was conducted in triplicates for each sample. The greater scavenging of DPPH free radicals was shown by a decreased absorbance of the solution. Ascorbic acid was taken as a standard reference, and the percentage of inhibition was computed as follows:

(9) $$\begin{array}{c} \text{ Inhibition }\left(\%\right)=\frac{\left(\text{AB}-\text{AS}\right)}{\text{AB}}\times 100, \end{array}$$

where AB is the absorption of a blank and AS is the absorption of a test sample.

To calculate the 50% inhibitory concentration (IC_50_) of each sample and ascorbic acid, the slope equation was utilized as follows:

(10) $$\begin{array}{c} Y=mx+c, \end{array}$$

where *m* is the slope, *x* is the concentration, and *c* is the intercept [35].

The technique followed by Amari et al. [34] was employed to determine the ferric-reducing antioxidant power (FRAP) of each sample. Stock solutions contained 300 mM acetate buffer (3.1 g C_2_H_3_NaO_2_·3H_2_O and 16 mL C_2_H_4_O_2_), pH 3.6, 10 mM 2,4,6-tripyridyl-s-triazine (TPTZ) solution in 40 mM HCl, and 20 mM FeCl_3_.6H_2_O. A new working solution was made by combining acetate buffer (25 mL), TPTZ solution (2.5 mL), and FeCl_3_.6H_2_O solution (2.5 mL). It was allowed to warm for about 37°C–40°C before use. Then, 0.3 mL of each sample extract at a concentration of 0.15 mg/mL was reacted with FRAP solution (3 mL) and distilled water (0.3 mL) in the dark for 30 min. Using a UV–Vis spectrophotometer, the absorbance of the reaction mixture was measured at 593 nm. The greater reducing power was shown by the higher absorbance of the mixture. The ascorbic acid equivalent was utilized to express the results, taking ascorbic acid as a positive reference. The experiment was conducted in triplicates for each sample.

### 2.9. Analysis of Phytochemical Constituents

The Folin–Ciocalteu technique was used to determine the total phenolic content of each sample, as stated in Agourram et al. [36] and Prakash et al. [37]. Briefly, each extract (150 *μ*L) was mixed with Folin–Ciocalteu reagent (1 mL) and 1 mL of sodium carbonate (75 g/L) in clean test tubes. For the purpose of developing color, the test tubes were vortexed and left in the dark for half an hour. A UV–Vis spectrophotometer was used to measure the solution's absorbance at 765 nm. Gallic acid was dissolved in absolute methanol at a concentration of 1.25 mg/mL to create a gallic acid standard solution. The milligrams of gallic acid equivalents (mg GAE) per gram of dry extract were used to express the total phenolic content. The experiment was conducted in triplicates for each sample.

The determination of the total flavonoid content was carried out following the methods previously used in Amari et al. [34]. In short, 1 mL of the methanolic extracts (0.125–0.15 mg/mL) was combined with 1 mL of 2% AlCl_3_ (w/v in distilled water). After 10 min of room temperature treatment, the mixture's absorbance was measured in relation to a blank at 415 nm. Quercetin standard (0–0.125 mg/mL) was used, and milligrams of quercetin equivalents (mg QE) per dry extract were employed to express the total flavonoid content in each sample. The experiment was done in triplicates for each sample.

The previous procedures used in Kumar et al. [38] were employed to determine the alkaloid content of each sample. In brief, a 10% acetic acid (v/v in ethanol) solution (50 mL) was used to dissolve 2 g of each sample powder. The solution was allowed to stand for 4 h after being shaken with a magnetic stirrer. Then, filtration was made using Whatman No. 1 filter paper, and the filtrate was evaporated on a hot plate to 1/4 of its original volume. To precipitate the alkaloids, a concentrated ammonium hydroxide solution was added dropwise to the resultant solution. The precipitate was washed with a 1% ammonium hydroxide solution after filtering it with a previously weighed Whatman No. 1 filter paper. After 2 h of drying at 60°C in a hot air oven, the filter paper containing alkaloid precipitates was cooled, followed by weight recording. The alkaloid content was ascertained gravimetrically from the weight difference using a triplicate analysis, and it was then quantified as a percentage of the sample examined.

### 2.10. Statistical Data Analysis

The results were reported using the mean and standard deviation. Tukey's honestly significant difference (Tukey's HSD) test was used to assess the mean differences at *p* < 0.05 using one-way analysis of variance (ANOVA). A paired sample *t*-test was also computed to compare the means at *p* < 0.05. Microsoft Excel (Version 2013) and Statistical Package for the Social Sciences (SPSS) Version 21.0 were used for the data analysis.

## 3. Results and Discussion

### 3.1. pH, Titratable Acidity, Vitamin C, and TSS

The investigated plants were significantly (*p* < 0.05) different in pH values and titratable acidity (Table 1). The fruits of *S. comorensis* and *S. guineense* subsp. *macrocarpum* had pH values of 3.52 ± 0.00 and 5.20 ± 0.01, respectively, and *D. praehensilis* tubers had pH values of 5.40 ± 0.02. The fruits of *S. comorensis* were most acidic (1.91% ± 0.02% citric acid/100 g), followed by the fruits of *S. guineense* subsp. *macrocarpum* (0.23% ± 0.01% citric acid/100 g), and the tubers of *D. praehensilis* were less acidic (0.06% ± 0.01% citric acid/100 g). The titratable acidity of fruits and vegetables extends their shelf-life by retaining their surface color and preventing microbial growth [39, 40]. However, titratable organic acids gradually decline through respiration during the storage of edible vegetables and fruits [41].

In *S. comorensis* fruits, the vitamin C concentration was 44.18 ± 0.50 mg AAE/100 g, but it was below the detection limit in *S. guineense* subsp. *macrocarpum* fruits. The fruit juices of *S. comorensis* and *S. guineense* subsp. *macrocarpum* had TSSs of 15.50 ± 0.56 and 7.10°Bx ± 0.36°Bx, respectively (Table 1). *S. comorensis* fruits had the highest vitamin C levels compared to the previously studied WEPs in southwestern [42] and northeastern Ethiopia [43], except for the leaves of *Vigna membranacea* A. Rich. and *Erucastrum abyssinicum* (A.Rich.) O.E.Schulz, which were found in those respective areas. Vitamin C is an essential water-soluble antioxidant that reduces the effect of oxidative stress [44] and promotes iron absorption in the human body [45]. Groups of compounds that dissolve in water, such as soluble sugars and other soluble substances, are included in TSSs [46]. In this respect, *S. comorensis* fruits had comparable TSS to most mango-based products, fulfilling the required ranges of TSS (14°Bx–28°Bx) and pH (3.5–4.0) [47]. This indicates the potential of *S. comorensis* fruits to be used, like mango fruits, in the form of various food products.

### 3.2. Proximate Composition

The study plants showed various levels of moisture, total ash, crude fiber, crude fat, crude protein, carbohydrate, and energy contents, as presented in Table 2. The total ash content ranged from 1.17 ± 0.29 g/100 g in *D. praehensilis* tubers to 2.25 ± 0.35 g/100 g in *S. comorensis* fruits. Crude fiber content was maximum in the fruits of *S. guineense* subsp. *macrocarpum* (15.50 ± 0.71 g/100 g) and *S. comorensis* (14.00 ± 1.41 g/100 g) and low in the *D. praehensilis* tubers (2.50 ± 0.71 g/100 g). The crude fat content in the fruit of *S. comorensis* was 4.00 ± 0.71 g/100 g, and it was found to be 0.75 ± 0.35 g/100 g in both *S. guineense* subsp. *macrocarpum* fruits and *D. praehensilis* tubers. The crude protein content ranged from 4.38 ± 0.17 g/100 g in *S. comorensis* fruits to 10.50 ± 0.18 g/100 g in *D. praehensilis* tubers. The utilizable carbohydrate content ranged from 59.63 ± 3.00 g/100 g in *S. comorensis* fruits to 68.83 ± 2.82 g/100 g in *D. praehensilis* tubers. The study plants contained calorific values ranging from 267.75 ± 5.56 kcal/100 g in *S. guineense* subsp. *macrocarpum* fruits to 324.08 ± 9.27 kcal/100 g in *D. praehensilis* tubers.

In the current study, lyophilized plant parts were almost similar (*p* > 0.05) in moisture content. The decrease in moisture content usually inhibits microbial development and enzymatic activity, keeping fruits and vegetables fresher for long periods of time [48]. The ash content in this study exceeds that of WEPs recorded by Wassie and Tsegay [49], although it was lower than the ash content of WEPs reported by other studies [22, 50–52] in Ethiopia. Ash content is often considered the amount of inorganic, macro, and essential minerals in fruits and vegetables [53]. The present study showed that *D. praehensilis* had the least crude fiber content among the investigated WEPs, in line with the previous study by Yimer et al. [22] in southwestern Ethiopia. In human health perspectives, soluble fibers can drop serum cholesterol levels, while insoluble fibers offer laxative advantages [54]. The crude fat content in *S. comorensis* fruits was comparable with the fruits of *Abelmoschus ficulneus* (L.) Wight & Arn. (4.00 ± 0.5 g/100 g) in the Metema and Quara districts of northwestern Ethiopia [51] and the leaves of *Solanum nigrum* (4.0 ± 0.6 g/100 g) and *V. membranacea* (4.3 ± 0.1 g/100 g) in southwestern Ethiopia [22]. On the other hand, the fat content in *S. guineense* subsp. *macrocarpum* and *D. praehensilis* was consistent with that of WEPs (0.12–0.9 g/100 g) in the Mekdela district of northern Ethiopia [55]. Moreover, the study conducted by Yiblet [55] reported low protein levels (0.98–3.22 g/100 g) from the fruit-bearing WEPs, similar to the current findings, whereas the previous study by Adamu et al. [43] reported maximum protein levels (13.10–33.63 g/100 g) from the green leaves and grain-bearing WEPs. The lower crude protein content in fruits might be linked with the existence of nonprotein nitrogen in senescent tissues and overripe fruits [56]. Carbohydrates are the most prevalent macronutrient in edible fruits and vegetables, ranging from 50% to 80% [56]. This confirms the abundance of carbohydrates in the studied plants. Consistent with the present findings, Yimer et al. [22] reported maximum gross energy (354.1 ± 5.4 kcal/100 g) from the tubers of *D. praehensilis*. Besides, the sweet fruits of *S. guineense* (Wild.) DC. subsp. *guineense* and *Carissa spinarum* L. were reported to have high energy values [7], similar to the wild edible fruits in the current finding. This indicates the essential role of wild fruits and vegetables as rich sources of energy for local communities.

### 3.3. Mineral Composition

The present study analyzed the calcium, copper, iron, magnesium, manganese, phosphorus, potassium, sodium, and zinc contents of the studied WEPs (Table 3). Calcium content ranged from 522.27 ± 12.23 mg/100 g in *S. comorensis* fruits to 995.04 ± 9.45 mg/100 g in *D. praehensilis* tubers. Both *S. comorensis* fruits and *D. praehensilis* tubers had 0.50 ± 0.04 mg/100 g copper content, while it was found to be below the detection limit in *S. guineense* subsp. *macrocarpum* fruits. The studied plants exhibited iron content ranging from 19.80 ± 1.31 mg/100 g in *S. comorensis* fruits to 111.94 ± 5.11 mg/100 g in *D. praehensilis* tubers. The magnesium content ranged from 923.25 ± 7.21 mg/100 g in *S. comorensis* fruits to 1592.18 ± 13.17 mg/100 g in *S. guineense* subsp. *macrocarpum* fruits. The manganese content ranged from 0.50 ± 0.06 mg/100 g in *S. guineense* subsp. *macrocarpum* fruits to 5.72 ± 0.21 mg/100 g in *D. praehensilis* tubers. The phosphorus content was significantly (*p* < 0.05) varied among the studied plants, ranging from 0.49 ± 0.01 mg/100 g in *D. praehensilis* tubers to 0.92 ± 0.01 mg/100 g in *S. comorensis* fruits. Potassium content ranged from 591.69 ± 7.99 mg/100 g in *D. praehensilis* tubers to 1357.71 ± 3.20 mg/100 g in *S. comorensis* fruits. Sodium content ranged from 0.60 ± 0.20 mg/100 g in *S. comorensis* fruits to 17.17 ± 0.40 mg/100 g in *S. guineense* subsp. *macrocarpum* fruits. The studied plants had zinc content ranging from 1.00 ± 0.03 mg/100 g in *D. praehensilis* tubers to 1.74 ± 0.10 mg/100 g in *S. guineense* subsp. *macrocarpum* fruits.

Root and tuberous vegetables were reported to contain a substantial level of calcium [45, 57], which is consistent with the current results. Besides, the calcium content in this study was comparable with that of WEPs (470.75–952.95 mg/100 g) from the Sedie Muja district of northern Ethiopia [49]. Calcium takes part in bone growth, tooth repair, and muscle contraction [58]. In line with the present findings, the previous studies [6, 22] reported very low concentrations of copper from WEPs in different parts of Ethiopia. In the current study, *D. praehensilis* tubers showed the highest amount of iron compared to fruit-bearing WEPs. This is in alignment with the report that root and tuberous vegetables were substantial in iron content [45, 57]. However, the iron level in *D. praehensilis* was relatively low (3.40 ± 0.10 mg/100 g) according to the study conducted in southwestern Ethiopia [22]. Iron plays vital roles in erythropoiesis, hemoglobin oxygenation, oxidation–reduction reactions, immune function, cell division and development, DNA synthesis, protein metabolism, thyroid hormone regulation, and neurotransmitter formation [59]. The amounts of magnesium in the studied plants were higher than the values, 68.20–588.10 mg/100 g [22], 56.65–72.79 mg/100 g [43], 87.96–473.24 mg/100 g [51], and 66.6–98.53 mg/100 g [52], previously reported from WEPs in Ethiopia. Dietary consumption of magnesium-rich plants reduces the frequency of obesity, Type 2 diabetes, and metabolic disorders [60]. The level of manganese in the present results was lower compared to in the WEPs (7.99–19.08 mg/100 g) recorded from northeastern Ethiopia [43]. Manganese was reported to play essential roles in the protection of impaired glucose metabolism and cardiometabolic health [61]. In the studied three WEPs, potassium was in higher concentration than sodium. Hence, the sodium-to-potassium ratio was less than one, similar to the previous reports [22, 62] in WEPs from other parts of Ethiopia. The zinc content in the current results was lower than the previous reports [22, 43, 49, 52] in different parts of Ethiopia. However, the lower zinc content (0.62 mg/100 g) in the fruits of *Ficus mucuso* Welw. ex Ficalho and *Dovyalis abyssinica* (A.Rich.) Warb. was reported by Jiru et al. [62] compared to the present findings. Zinc is essential for the growth, differentiation, and apoptosis of cells, in addition to its role in the immune system, energy production, hemostasis, thrombosis, repair, reproduction, vision, taste, neurogenesis, and wound healing [59, 63].

### 3.4. Antinutritional Factors

The current results showed that the level of phytic acid ranged from 65.11 ± 1.44 mg/100 g in *S. comorensis* fruits to 70.67 ± 1.68 mg/100 g in *S. guineense* subsp. *macrocarpum* fruits. The amount of oxalic acid ranged from 170.00 ± 20.00 mg/100 g in *S. guineense* subsp. *macrocarpum* fruits to 790.00 ± 110.00 mg/100 g in *S. comorensis* fruits. The tannin content ranged from 196.51 ± 4.93 mg CE/100 g in *D. praehensilis* tubers to 11,147.55 ± 139.26 mg CE/100 g in *S. comorensis* fruits (Table 4).

The amount of phytic acid in the studied plants was higher than the previous reports, 0.02–0.17 mg/100 g [64], 0.33–1.52 mg/100 g [55], and 0.85–1.85 mg/100 g [50], on the phytate content of WEPs. However, it was exceeded by the levels of phytic acid, 80.78–168.99 mg/100 g [51] and 103.67–197.74 mg/100 g [52], reported from WEPs in other parts of Ethiopia. In addition, our results were lower than the levels of phytic acid (175.60–307.30 mg/100 g) in the leaves of *Cleome gynandra* L., *S. nigrum* L., and *V. membranacea* from southwestern Ethiopia [22]. The study plants were also higher in oxalate content relative to the reports, 3.37–11.73 mg/100 g [64], 0.52–0.92 mg/100 g [55], 0.46–1.7 mg/100 g [50], and 13.35–48.42 mg/100 g [52] in the earlier studies. However, a study conducted by Tadesse et al. [51] has reported the maximum levels of oxalate (609.29–945.45 mg/100 g) from WEPs in northwestern Ethiopia. Similarly, findings by Yimer et al. [22] revealed that the leaves of *C. gynandra* (205.00 mg/100 g), *S. nigrum* (443.90 mg/100 g), and *V. membranacea* (307.30 mg/100 g) had more oxalate than the current plants, except for the fruits of *S. comorensis*. The sweetly sour taste of *S. comorensis* fruits might be due to the high oxalic acid concentration, which induces a sharp acidic taste [65]. It was indicated that diets high in phytic acid and oxalates resulted in low bioavailability of calcium, causing calcium deficiency diseases like rickets and osteomalacia [66].

The amount of tannins in the studied plants was much greater than that in the previous reports by Adamu et al. [64] (1.38–5.49 mg/100 g), Rumicha et al. [52] (6.73–25.95 mg/100 g), Wassie [50] (0.79–1.57 mg/100 g), and Yiblet [55] (0.23–0.53 mg/100 g). However, the level of tannins (6314 mg/100 g) in *Ximenia caffra* Sond. revealed by Getachew et al. [6] is higher than the present reports, except for *S. comorensis* fruits. Consistent with the current findings, it was reported that higher levels of tannins are found in edible fruit parts than in tubers [6, 51]. The highest tannin content might be very crucial in medicinal perspectives owing to the antioxidant role of tannic acid [67], although it affects the absorption of nutrients in the human body [19, 68]. The phytic acid, oxalic acid, and tannins in *D. praehensilis* tubers were higher in the current findings relative to a similar study conducted on the same plant [22]. The variation in the antinutritional levels might be because of the genetic variations or the differences in the edaphic (soil) factors where the plant samples were collected. Since antinutritional factors pose adverse effects on nutrient bioavailability, certain food preparation approaches like fermentation, cooking, puffing, and soaking can be employed to reduce their amount in edible plants [19].

### 3.5. Antioxidant Activity

#### 3.5.1. DPPH Free Radical Scavenging Activity

**Figure 2:**
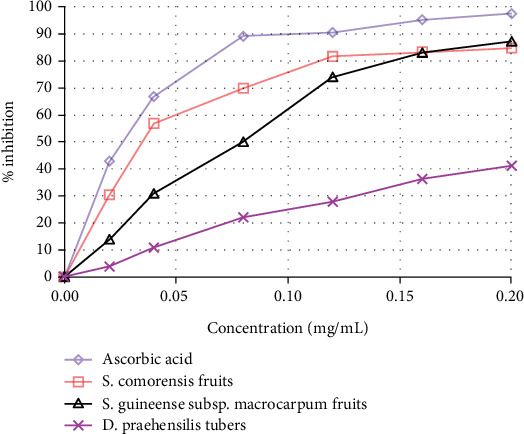
DPPH free radical scavenging activity of *S. comorensis* fruits, *S. guineense* subsp. *macrocarpum* fruits, and *D. praehensilis* tubers.

The DPPH free radical scavenging capacity of the three studied WEPs is shown in Figure 2. Accordingly, as the concentration of extracts increased, so did their capacity to scavenge free radicals. With the corresponding concentrations (0.00, 0.02, 0.04, 0.08, 0.12, 0.16, and 0.20 mg/mL), the methanolic extracts of *S. comorensis* fruits had the highest (%inhibition = 0.00, 30.60, 56.88, 69.96, 81.79, 83.36, and 84.71) DPPH free radical scavenging ability, followed by the extracts of *S. guineense* subsp. *macrocarpum* fruits (%inhibition = 0.00, 13.98, 31.15, 50.20, 74.32, 83.27, and 87.39), whereas extracts of *D. praehensilis* tubers exhibited the lowest DPPH free radical scavenging activity (%inhibition = 0.00, 4.13, 10.93, 22.07, 27.85, 36.26, and 41.24).

Compared to the previous findings by Adamu et al. [64], the fruits of *S. comorensis* and *S. guineense* subsp. *macrocarpum* exhibited comparability with the leaves of *E. abyssinicum* and *Haplocarpha schimperi* Beauverd, respectively, in DPPH free radical scavenging ability at the same concentration (0.20 mg/mL). It was realized that antioxidants decrease DPPH free radicals by providing electrons and creating stable compounds, for example, 2,2-diphenyl-1-hydrazine [69]. Free radicals like reactive oxygen species damage biomolecules, leading to oxidative stress at higher concentrations [70]. Antioxidant phenolic compounds have the ability to scavenge free radicals and prevent the oxidation of cellular components [71]. Hence, they reduce the incidence of chronic illnesses like diabetes, cardiovascular and gastrointestinal disorders, and some types of cancer [70]. Antioxidant molecules from plants include phytochemicals such as phenolics, flavonoids, flavonols, tannins, saponins, proanthocyanidins, and *α*-glucosidase [71].

**Figure 3:**
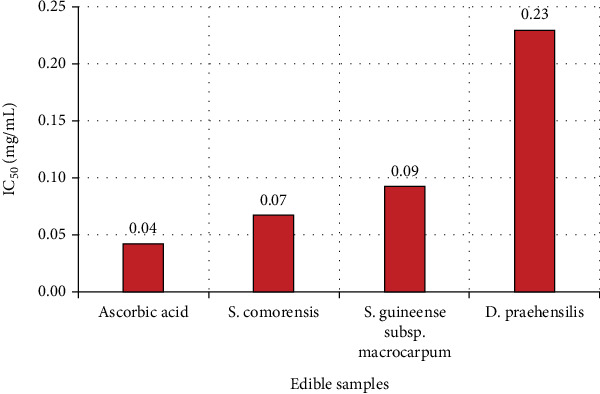
IC_50_ values in scavenging DPPH free radicals by *S. comorensis* fruits, *S. guineense* subsp. *macrocarpum* fruits, and *D. praehensilis* tubers.

The lowest concentration of plant extracts required to prevent 50% of free radicals in DPPH is presented in Figure 3. Accordingly, extracts of *S. comorensis* and *S. guineense* subsp. *macrocarpum* fruits exhibited substantial antioxidant capacity, with respective IC_50_ values of 0.07 and 0.09 mg/mL, compared to ascorbic acid (IC_50_ = 0.04 mg/mL), whereas the lowest antioxidant potential was observed in extracts of *D. praehensilis* tubers (IC_50_ = 0.23 mg/mL). Similar to the current findings, Yimer et al. [42] revealed that *D. praehensilis* tubers had the least antioxidant capacity among the studied WEPs in southwestern Ethiopia. This implies that *D. praehensilis* tubers are insignificant in terms of antioxidant activity, though they are nutritionally rich relative to certain WEPs.

#### 3.5.2. FRAP

The antioxidant activity of the three WEPs was also measured using the ferric-reducing power of the methanolic extracts (Table 5). The highest ferric-reducing power was obtained in the extracts of *S. guineense* subsp. *macrocarpum* fruits (133.00 ± 4.15 mg AAE/100 g extract) (*p* < 0.05) compared to the extracts of *S. comorensis* fruits (90.10 ± 5.03 mg AAE/100 g extract) and *D. praehensilis* tubers (85.31 ± 0.73 mg AAE/100 g extract), with a concentration of 0.15 mg/mL.

### 3.6. Phytochemical Constituents

Phytochemicals such as phenolics, flavonoids, and alkaloids were significantly (*p* < 0.05) different among the edible parts of the studied plants (Table 6). The total phenolic content was the highest in extracts of *S. comorensis* fruits (285.01 ± 5.77 mg GAE/100 g), and it was found to be the lowest in extracts of *D. praehensilis* tubers (73.81 ± 1.10 mg GAE/100 g). The extracts of *S. comorensis* fruits had also the highest total flavonoids (241.87 ± 4.37 mg QE/100 g), while extracts of *D. praehensilis* tubers exhibited the lowest (124.97 ± 5.35 mg QE/100 g). The alkaloids content ranged from 2000.00 ± 710.00 mg/100 g in *D. praehensilis* tubers to 6500.00 ± 710.00 mg/100 g in *S. comorensis* fruits.

The total phenolics in the studied plants were higher compared to the values (0.79–17.02 mg GAE/100 g) reported by Adamu et al. [64] in WEPs collected from northeastern Ethiopia. Additionally, the total phenolic content of *S. comorensis* fruits was maximum compared to the phenolic content (108.32–230.76 mg GAE/100 g) in WEPs reported by Jiru et al. [62] from the East Wollega zone, western Ethiopia. However, the total phenolics in the studied plants were lower than the values (2297.00–3573.00 mg GAE/100 g) in the leaves of *C. gynandra*, *S. nigrum*, *Trilepisium madagascariense* DC., and *V. membranacea* collected from southwestern Ethiopia [42]. It has been suggested that plant-based phenolics have antioxidant benefits and prevent a number of chronic illnesses [64, 72]. The total flavonoids in the studied plants were much greater than the values (2.27–7.12 mg QE/100 g) in WEPs reported by Adamu et al. [64] from northeastern Ethiopia. Flavonoids modulate intracellular signaling cascades, which are essential to cellular functions, in addition to their antioxidant properties [73]. The total alkaloids in the studied plants were comparable with the alkaloid content (1.59–6.37 g/100 g) of WEPs reported in northern Uganda [74]. The plant-derived alkaloids are reported to have a healing ability for myotonic dystrophy Type I [75]. Due to the fact that different plant species may produce various levels of secondary metabolites, the phytochemical constituents are varied among different WEP species. Therefore, the investigated WEPs will have various antioxidant and therapeutic potentials in addition to their nutritional importance.

## 4. Conclusions

In this study, *S. comorensis* fruits were significant in titratable acidity, TSS, and vitamin C. The fruits of both *S. comorensis* and *S. guineense* subsp. *macrocarpum* were rich in crude fiber content. Overall, the three WEPs were appreciated for their carbohydrate content and calorific values. The studied plants were also chief sources of minerals like calcium, magnesium, iron, and potassium. The amount of phytic acid was optimal in the three investigated plants, while the levels of oxalic acid and tannins were found to be high in *S. comorensis* fruits. The fruits of *S. comorensis* and *S. guineense* subsp. *macrocarpum* were observed to have substantial levels of phytochemicals, which are essential to their antioxidant activities. Thus, they showed significant (*p* < 0.05) DPPH free radical scavenging ability and ferric-reducing power, respectively. In general, the studied WEPs are critical in nutritional and pharmaceutical perspectives and need to be conserved and widely used by the local communities as well as the people in the country as a whole.

### 4.1. Limitation of the Study and Future Suggestion

Due to the limited financial access and study time restriction, this study was conducted only on the three commonly consumed WEPs, among others. Therefore, other studies will be required to address the nutritional, antinutritional, antioxidant, and phytochemical analysis of the rest of the most commonly edible wild plants in the Dibatie district, western Ethiopia. Additionally, because of the limited access to laboratory equipment and chemicals, certain parameters such as toxicity studies, carotenoid content, and fatty acid content are not evaluated for each of the three WEPs. Hence, further investigations will be crucial to examine the remaining parameters in the future.

## Figures and Tables

**Table 1:** pH value, titratable acidity, vitamin C, and TSS of WEPs, *S. comorensis* fruits, *S. guineense* subsp. *macrocarpum* fruits, and *D. praehensilis* tubers.

**Parameters**	**Plant species**		
	***S. comorensis***	***S. guineense* subsp. *macrocarpum***	***D. praehensilis***
pH value	3.52 ± 0.00^c^	5.20 ± 0.01^b^	5.40 ± 0.02^a^
% Acidity (% citric acid/100 g)	1.91 ± 0.02^a^	0.23 ± 0.01^b^	0.06 ± 0.01^c^
Vitamin C (mg AAE/100 g)	44.18 ± 0.50	BDL	ND
Total soluble solids (°Brix)	15.50 ± 0.56^∗^	7.10 ± 0.36	ND

*Note:* Values are the mean ± standard deviation (SD) of triplicate analyses. Values with different superscripts within a row are different (*p* < 0.05) using one-way analysis of variance (ANOVA), Tukey's test.

Abbreviations: AAE, ascorbic acid equivalent; BDL, below detection limit; ND, not determined.

⁣^∗^Significant difference (*p* < 0.05) between mean values using paired *t*-test.

**Table 2:** Proximate composition of WEPs, *S. comorensis* fruits, *S. guineense* subsp. *macrocarpum* fruits, and *D. praehensilis* tubers.

**Nutrients**	**Plant species**		
	***S. comorensis***	***S. guineense* subsp. *macrocarpum***	***D. praehensilis***
Moisture fresh weight (g/100 g)	77.42 ± 0.76^b^	82.24 ± 1.66^a^	71.15 ± 2.02^c^
Moisture dry weight (g/100 g)	15.75 ± 0.35^a^	16.50 ± 0.00^a^	16.25 ± 1.77^a^
Total ash (g/100 g)	2.25 ± 0.35^a^	2.00 ± 0.50^ab^	1.17 ± 0.29^b^
Crude fiber (g/100 g)	14.00 ± 1.41^a^	15.50 ± 0.71^a^	2.50 ± 0.71^b^
Crude fat (g/100 g)	4.00 ± 0.71^a^	0.75 ± 0.25^b^	0.75 ± 0.25^b^
Crude protein (g/100 g)	4.38 ± 0.17^c^	5.60 ± 0.18^b^	10.50 ± 0.18^a^
Carbohydrate (g/100 g)	59.63 ± 3.00^b^	59.65 ± 1.28^b^	68.83 ± 2.82^a^
Energy (kcal/100 g)	292.00 ± 4.95^b^	267.75 ± 5.56^c^	324.08 ± 9.27^a^

*Note:* Values are the mean ± SD of triplicate analyses. Values with different superscripts within a row are different (*p* < 0.05) using one-way ANOVA, Tukey's test.

**Table 3:** Mineral composition of WEPs, *S. comorensis* fruits, *S. guineense* subsp. *macrocarpum* fruits, and *D. praehensilis* tubers.

**Minerals**	**Plant species**		
	***S. comorensis***	***S. guineense* subsp. *macrocarpum***	***D. praehensilis***
Calcium (mg/100 g)	522.27 ± 12.23^c^	919.09 ± 11.12^b^	995.04 ± 9.45^a^
Copper (mg/100 g)	0.50 ± 0.04^a^	BDL	0.50 ± 0.04^a^
Iron (mg/100 g)	19.80 ± 1.31^b^	22.36 ± 2.06^b^	111.94 ± 5.11^a^
Magnesium (mg/100 g)	923.25 ± 7.21^c^	1592.18 ± 13.17^a^	1044.79 ± 8.14^b^
Manganese (mg/100 g)	1.73 ± 0.11^b^	0.50 ± 0.06^c^	5.72 ± 0.21^a^
Phosphorus (mg/100 g)	0.92 ± 0.01^a^	0.54 ± 0.00^b^	0.49 ± 0.01^c^
Potassium (mg/100 g)	1357.71 ± 3.20^a^	1094.83 ± 3.39^b^	591.69 ± 7.99^c^
Sodium (mg/100 g)	0.60 ± 0.20^b^	17.17 ± 0.40^a^	16.98 ± 0.20^a^
Zinc (mg/100 g)	1.73 ± 0.10^a^	1.74 ± 0.10^a^	1.00 ± 0.03^b^

*Note:* Values are the mean ± SD of triplicate analyses. Values with different superscripts within a row are different (*p* < 0.05) using one-way ANOVA, Tukey's test.

Abbreviation: BDL, below detection limit.

**Table 4:** Antinutritional constituents of WEPs, *S. comorensis* fruits, *S. guineense* subsp. *macrocarpum* fruits, and *D. praehensilis* tubers.

**Antinutritional compounds**	**Plant species**		
	***S. comorensis***	***S. guineense* subsp. *macrocarpum***	***D. praehensilis***
Phytic acid (mg/100 g)	65.11 ± 1.44^b^	70.67 ± 1.68^a^	69.88 ± 0.03^a^
Oxalic acid (mg/100 g)	790.00 ± 110.00^a^	170.00 ± 20.00^b^	190.00 ± 20.00^b^
Tannins (mg CE/100 g)	11,147.55 ± 139.26^a^	6094.11 ± 189.77^b^	196.51 ± 4.93^c^

*Note:* Values are the mean ± SD of triplicate analyses. Values with different superscripts within a row are different (*p* < 0.05) using one-way ANOVA, Tukey's test.

Abbreviation: CE, catechin equivalent.

**Table 5:** Ferric-reducing capacity of WEPs, *S. comorensis* fruits, *S. guineense* subsp. *macrocarpum* fruits, and *D. praehensilis* tubers.

**Wild edible plant species**	**FRAP at 0.15 mg/mL**
	**mg AAE/100 g extract**
*S. comorensis*	90.10 ± 5.03^b^
*S. guineense* subsp. *macrocarpum*	133.00 ± 4.15^a^
*D. praehensilis*	85.31 ± 0.73^b^

*Note:* Values are the mean ± SD of triplicate analyses. Values with different superscripts within a column are different (*p* < 0.05) using one-way ANOVA, Tukey's test.

Abbreviation: AAE, ascorbic acid equivalent.

**Table 6:** Phytochemical constituents of WEPs, *S. comorensis* fruits, *S. guineense* subsp. *macrocarpum* fruits, and *D. praehensilis* tubers.

**Phytochemical compounds**	**Plant species**		
	***S. comorensis***	***S. guineense* subsp. *macrocarpum***	***D. praehensilis***
Total phenolics (mg GAE/100 g dry extract)	285.01 ± 5.77^a^	174.66 ± 7.22^b^	73.81 ± 1.10^c^
Total flavonoids (mg QE/100 g dry extract)	241.87 ± 4.37^a^	168.65 ± 4.26^b^	124.97 ± 5.35^c^
Total alkaloids (mg/100 g dry sample)	6500.00 ± 710.00^a^	4750.00 ± 350.00^b^	2000.00 ± 710.00^c^

*Note:* Values are the mean ± SD of triplicate analyses. Values with different superscripts within a row are different (*p* < 0.05) using one-way ANOVA, Tukey's test.

Abbreviations: GAE, gallic acid equivalent; QE, quercetin equivalent.

## Data Availability

The data that support the findings of this study are available on request from the corresponding author.
